# Widefield Optical Coherence Tomography Angiography in Diabetic Retinopathy

**DOI:** 10.1155/2020/8855709

**Published:** 2020-11-24

**Authors:** Alessia Amato, Francesco Nadin, Federico Borghesan, Maria Vittoria Cicinelli, Irini Chatziralli, Saena Sadiq, Rukhsana Mirza, Francesco Bandello

**Affiliations:** ^1^Department of Ophthalmology, IRCCS San Raffaele Scientific Institute, Milan, Italy; ^2^School of Medicine, Vita-Salute San Raffaele University, Milan, Italy; ^3^Department of Ophthalmology, Feinberg School of Medicine, Northwestern University, Chicago, IL, USA; ^4^2nd Department of Ophthalmology, National and Kapodistrian University of Athens, Athens, Greece

## Abstract

**Purpose:**

To summarize the role of widefield optical coherence tomography angiography (WF-OCTA) in diabetic retinopathy (DR), extending from the acquisition strategies to the main clinical findings.

**Methods:**

A PubMed-based search was carried out using the terms “Diabetic retinopathy”, “optical coherence tomography angiography”, “widefield imaging”, and “ultra-widefield imaging”. All studies published in English up to August 2020 were reviewed.

**Results:**

WF-OCTA can be obtained with different approaches, offering advantages over traditional imaging in the study of nonperfusion areas (NPAs) and neovascularization (NV). Quantitative estimates and topographic distribution of NPA and NV are useful for treatment monitoring and artificial intelligence-based approaches. Curvature, segmentation, and motion artifacts should be assessed when using WF-OCTA.

**Conclusions:**

WF-OCTA harbors interesting potential in DR because of its noninvasiveness and capability of objective metrics of retinal vasculature. Further studies will facilitate the migration from traditional imaging to WF-OCTA in both the research and clinical practice fields.

## 1. Introduction

Diabetes mellitus (DM) is a major public health concern, with a global prevalence expected to rise from 8.8% in 2015 to 10.4% in 2040 [1]. Diabetic retinopathy (DR) is a microangiopathic complication of DM and, despite the latest diagnostic and therapeutic advancements, is still one of the leading causes of blindness worldwide, affecting about one-third of diabetic patients [2–6]. Considering its burden, efficient management of DR patients depends on proper classification and severity grading which, in turn, lay the foundations on the most appropriate imaging modality.

Until relatively recent times, the classification of DR has been relying on stereoscopic color fundus photography (CFP); the Early Treatment Diabetic Retinopathy Study (ETDRS) grading system, a 13-level severity scale based on 7-field photography ranging from early changes to severe proliferative DR (PDR), has been the gold standard for years. Fundus fluorescein angiography (FFA) is another useful tool for the classification of DR, as it allows the detection of the blood-retinal barrier (BRB) breakdown, microaneurysms (MA), nonperfusion areas (NPAs), intraretinal microvascular abnormalities (IRMA), and neovascularization (NV). FFA is relatively invasive, and it might be associated with the risk of life-threatening allergic reactions to the intravenous dye. It is relatively contraindicated in the case of kidney disease, which is fairly common in diabetic patients, and pregnancy. FFA does not allow for a separate visualization of the different retinal vascular plexuses. Lastly, the FFA-based assessment of DR severity mainly relies on a qualitative approach.

With the introduction of optical coherence tomography angiography (OCTA) technology, a turning point was set in the management of DR [7–10]. OCTA is an OCT-derived technique that generates high-resolution angiographic images by using repeated B-scans to detect motion contrast from flowing erythrocytes. Currently available OCTA technologies acquire clusters of 2-4 B-scans on the “*x*” fast axis at each of the “*y*” slow scan axis points, and the software eventually extracts the moving image from each of such clusters. OCTA offers several advantages over traditional angiography, including avoidance of dye injection and the provision of depth-resolved information of the superficial and deep retinal vasculature. OCTA image resolution is higher compared to FFA and allows quantitative data processing, including measurement of vessel density (VD), vessel length density (VLD), perfusion density (PD), and the size and the shape of the foveal avascular zone (FAZ) [7, 11].

The OCTA field of view has been limited to 6 × 6 mm scans until recent times. Efforts to obtain a wider field of view have been pursued with increasing success; the results achieved have ushered in a new era in disease management. Widefield (WF) OCTA imaging represents the state of the art in retinal vascular diseases, including DR. This novel imaging technique has offered great advantages in the detection of NPAs and NV. The present review is aimed at summarizing the role of WF-OCTA in DR, extending from the acquisition strategies to the main clinical findings.

## 2. Technical Solutions to Achieve WF-OCTA

The WF-OCTA scan can be obtained with three main approaches:

- Extended field imaging (EFI), which consists of the employment of trial frames fitted with a magnifying +20 D lens
- Montage technique, including a variety of protocols aimed at merging multiple smaller scans
- Single-shot widefield 12 × 12 mm scans or 15 × 9 mm scans

### 2.1. EFI

The interposition of a positive diopter lens between the OCTA probe and the eye results in an increased light incidence angle and thus an imaging field expansion [12].

Uji and Yoshimura compared the scan length measurements obtained with conventional imaging (spectral-domain (Spectralis OCT, Spectralis HRA + OCT, Heidelberg Engineering, Heidelberg, Germany) and a swept-source OCT (DRI OCT-1, Topcon, Tokyo, Japan)) with those of EFI-OCT applied to the two OCT systems. They reported a statistically significant difference in both the horizontal and the vertical direction, with a scan length corresponding to the nearly 60-70° angle field when EFI was coupled with DRI OCT-1 [12].

When the EFI technique has been applied to SS-OCTA (Plex® Elite 9000, Carl Zeiss Meditec, Dublin, California, USA), it resulted in a larger scan size than both SS-OCTA images acquired without EFI and traditional HRA2 FFA captured using a 55° Heidelberg lens. In eyes with DR, EFI-SS-OCTA had good sensitivity (96% and 79%, respectively) and specificity (100% and 96%, respectively) in detecting NPA and NV compared to FFA. WF-OCTA with EFI was significantly more comfortable for patients than conventional dye angiography [13]. Parallelly, Pellegrini et al., using a similar study design, evaluated the extension of NPA and the presence and the number of NV. Aside from a larger captured fundus area, EFI SS-OCTA revealed a significantly larger extension of NPA compared to non-EFI SS-OCTA and FFA [14].

EFI is a simple and economic technique that can be carried out with readily available equipment. However, it covers a larger retinal territory with the same number of A-scans of non-EFI acquisitions; i.e., it extends the field of the image by magnifying each pixel, reducing the global slab resolution. This may lead to underestimation of VD and overestimation of NPA in DR evaluation; validation of this technique with regard to traditional imaging is still warranted.

### 2.2. Montage Technique

The montage technique consists of assembling multiple smaller scans to gather a wider OCTA image, while maintaining an adequate axial and lateral resolution. A variety of montage protocols have been adopted.

de Carlo et al. were among the first to use this approach to improve the visualization of the retinal vasculature in a small prospective case series [15]. The authors included one patient with unilateral branch retinal vein occlusion, one patient with bilateral severe PDR, and one healthy control. By combining nine adjacent 3 × 3 mm OCTA scans (AngioVue XR Avanti, Optovue, Fremont, CA), the authors managed to create a single WF montage OCTA of approximately 8 × 8 mm or 30° field, without moving the patient's fixation point. The montage OCTA showed the retinal vasculature in the greatest details compared to a single 8 × 8 mm OCTA scan acquired using 304 × 304 A-scans. Once compared to 50° FFA, montage OCTA identified more pathology than FFA or single-scan 8 × 8 mm OCTA.

More recently, Lavia et al. got an advantage from the high resolution provided by the 3 × 3 mm OCTA scans of the 100 kHz PlexElite 9000 SS-OCTA to obtain WF images of the retina in healthy individuals [16]. The authors acquired an average of 25 3 × 3 mm scans from the fovea to the retinal periphery to create a horizontal and a vertical band passing through the fovea. The scanning cursor was manually moved to the desired area ensuring an overlay of about 40% between adjacent volumes. Afterward, consecutive 3 × 3 mm volumes were manually superimposed using a single 12 × 12 mm scan as a reference. This method was able to limit low-signal artifacts, which are a major drawback of larger scans, and allowed for better segmentation and quantitative analyses.

Montage protocols are not rigidly standardized and can be customized by the operators. In a small prospective study aimed at quantifying the burden of microvascular disease in the eyes with PDR, Zhang et al. captured a 100-degree field of view using sixteen 6 × 6 mm scans. Montage WF-OCTA images provided a more accurate visualization of the retinal vascular plexuses than the traditional FFA, showing a higher burden of pathology in the retinal periphery [17].

Though allowing for high-quality images, the montage technique is time-consuming and labor-intensive, requiring optimal patients' cooperation to avoid misalignment; this may hinder the acquisition in subjects with low visual acuity and poor fixation. Due to these drawbacks, montage WF-OCTA is currently hard to export from the field of research to the clinical setting [17].

### 2.3. Single-Shot Scans

The newest generation of SS-OCTA is embedded with one-shot WF acquisition of 15 × 9 mm or 12 × 12 mm scans, corresponding to a 40° field of view [18], which can be further combined into greater composite images up to 80° of the retina [19, 20]. Single-shot acquisitions are faster to obtain compared to montage scans and provide information of the entire posterior pole, though sacrificing to some extent the image resolution.

Hirano et al. evaluated the vascular morphology in patients with varying severities of DR using different SS-OCTA image sizes and reported that 3 × 3 mm images had the best diagnostic performance in predicting DR eyes from a pool of healthy controls. The superior sensitivity of 3 × 3 mm macular scans for the detection of DR vascular abnormalities might seem contradictory at a first glance, since DR primarily affects the retinal periphery.

The lower resolution of larger scans as compared to a smaller field of view may partially explain these findings [21, 22]. Moreover, as the image size increases, so does the proportion occupied by larger blood vessels; as the pathogenesis of DR is dominated by microvascular damage, larger scans can occult or underestimate the entity of small vessel dropout [21]. Large vessel removal from VD quantification on WF-OCTA images has yielded improved diagnostic performance in terms of discriminating different stages of DR [23]. Some studies reported that the DR-related microvascular damage begins in the perimacular area (as suggested by the FAZ enlargement often observed in DR patients) [24], making the 3 × 3 mm scan the one with the best predictive value in DR. Finally, the 12 × 12 and 15 × 9 mm scans harbor peripheral and motion artifacts that can interfere with quantitative and qualitative analyses of the retinal vasculature [25].

WF-OCTA artifacts in DR patients fall into 3 categories: systemic artifacts (i.e., projection artifacts, masking, unmasking, and loss of signal), image processing errors (i.e., segmentation, duplication of vessels, and alignment errors), and motion artifacts (i.e., displacement, blink artifacts, and stretch artifacts). Because of the large dimension of the scans, the time necessary for acquisition, and the automated fusion operated by the instrument, OCTA montage images are susceptible to all these types of artifacts. Patients with higher-severity DR eyes with NV, epiretinal membrane, diabetic macular edema, and pigment epithelium detachment may be at particular risk for poor-quality imaging [26].

## 3. WF-OCTA and Nonperfusion Areas in DR

**Figure 1:**
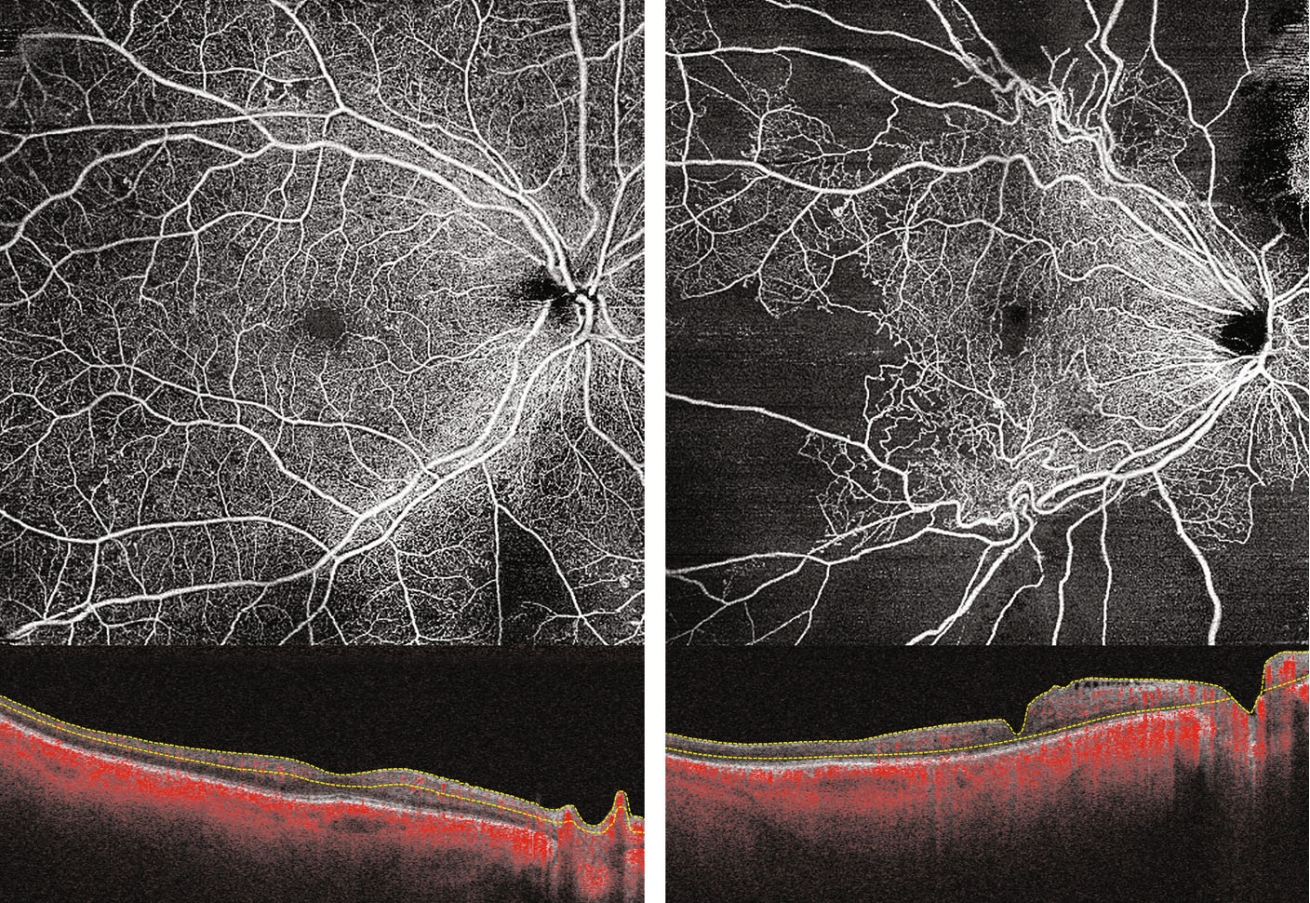
Two patients with diabetic retinopathy showing different degrees of capillary nonperfusion.

Capillary occlusion is the pivotal mechanism in DR progression [27–30]. Peripheral nonperfusion has been associated with visual deterioration and visual field damage progression (Figure 1) [31]. The quantification of the extent of retinal ischemia might be a promising prognostic biomarker in DR, aiding in tailoring personalized treatment algorithms [32, 33].

### 3.1. Diagnostic Performance of WF-OCTA in NPA Detection

WF-OCTA has very high sensitivity and discretely high specificity for NPA detection, using UWF-FFA as a reference [34]. WF-OCTA 12 × 12 mm scans demonstrated good discriminating accuracy in classifying eyes with DR (any severity) versus diabetic eyes without retinopathy, with an area under the curve (AUC) of 0.93 [35]. When nonproliferative DR (NPDR) eyes were compared to diabetic eyes without retinopathy, OCTA yielded poorer results (AUC was 0.875).

As capillary dropout in NPDR initially occurs in the midperiphery, wider scans are necessary to accurately determine the severity of DR, provided that peripheral NVs are carefully excluded as they can alter quantitative vascular metrics. Tan et al. [23] compared the diagnostic accuracy of the 12 × 12 mm with a 6 × 6 mm OCTA image. The authors cropped a 12 × 12 mm image into a central 6 × 6 mm field and a peripheral square annulus region and calculated four different vascular metrics (namely, the total perfusion density (TPD), the capillary perfusion density (CPD), the large vessel density (LVD), and the capillary dropout density (CDD)) in each subregion. They found a stepwise increase in CDD from no DR to severe DR; the CCD in the peripheral square annulus was the best parameter discriminating between mild NPDR and no DR groups. The TPD and CPD in the peripheral subfield had higher discriminative power for more advanced stages of DR than small-field images.

### 3.2. Further Characterization of Vascular Anatomy in DR

The introduction of projection-resolved (PR) OCTA algorithms [36, 37] and 3D visualization [38] systems has increased the quality of depth-resolved OCTA scans. Indeed, while in 2D reconstructions, retinal layers are segmented and reconstructed by two topographic axes (i.e., *x* and *y*), volume-rendered OCTA reconstructions integrate structural and angiographic data to create 3D images. By incorporating the *z*-axis to the OCTA slabs, the relationship between retinal vascular and morphological alterations can be further investigated, e.g., the association between flow voids and cystoid spaces in DME [39] or between flow voids and areas of disorganization of the retinal inner layers [40]. 3D OCTA, implemented with a rotational display mode, may also assess the distribution of microaneurysms in relation to macular ischemia [41].

The exact distribution of the capillary plexuses in the mid-to-far periphery has not been investigated to a similar extent. In healthy eyes, the capillary density of the intermediate capillary plexus (ICP) and the deep capillary plexus (DCP) progressively reduces from the fovea to the periphery, with the ICP ultimately disappearing at about 8-9 mm of eccentricity. Parallelly, the ganglion cell complex and the inner nuclear layer thin with a centrifugal fashion [16].

Diabetic microangiopathy starts in the midperiphery and extends towards the perifoveal region with progressive severity of the disease [42]. Capillary dropout displays a sectorial preference for the temporal quadrants, at least in the early stages of NPDR. The rarefaction of the vascular supply in the midperiphery (where the vascular plexuses merge from 3 into 2 networks) and the presence of radial peripapillary capillaries in the nasal quadrant, together with decreased retinal thickness temporally, might contribute to the topographic distribution of NPA in diabetic eyes [43, 44]. The macular area bears a less amount of NPAs as compared to the midperiphery, thanks to multiple overlappingly plexa into the superficial and the deeper retinal layers.

Yasukura and associates hypothesized the existence, in the extramacular region, of distinct lobules of retinal perfusion, separated by each other by large arterioles encompassing both the superficial and deep layers [45]. They also posed that extramacular retinal areas could be divided into two groups: those perfused by a singular arteriolar trunk and those dually nourished. By using 12 × 12 mm OCTA slabs, the authors found higher vulnerability of areas nourished by a single arteriole to diabetic microangiopathy. There were no differences in the extramacular NPAs between severe NPDR and PDR, while the eyes with PDR had significantly greater NPAs in the macular area than those with severe NPDR. The importance of the different vascular anatomic configurations between the macular and extramacular regions may have other clinical correlates, including the distribution and the extent of cotton wool spots [46]. Extramacular cotton wool spots (or white spots) have been mostly associated with NPAs encompassing all retinal layers, as opposed to macular cotton wool spots, which are more associated with NPAs in the superficial layer only [46].

Preferential localization of NPAs along the main retinal arteries has also been described [47]. Ishibazawa et al. examined 63 eyes from 44 patients with NPDR or PDR aided by a computer-based algorithm determining the shortest distance between nonperfusion and retinal vessels. The authors found a larger rate of arterial-adjacent NPAs compared with venous-adjacent NPAs in all stages of DR. The authors hypothesized diabetic microangiopathy starting near the arterial side, with no regard to the level of DR severity, and then progressing towards the venous side.

Finally, WF-OCTA demonstrated more pronounced vascular involvement in the DCP, irrespective of the stage of DR. A recent study of 104 eyes with PDR showed a disproportion of lamellar capillary nonperfusion between the SCP and the DCP, the latter being more greatly involved in all the assessed quadrants [48]. The difference in the perfusion pressure between the SCP and the DCP could be a plausible explanation of this discrepancy [49].

In light of the above-described findings, OCTA provides additional clues of the vascular involvement in diabetic microangiopathy, as well as a quantitative estimate and topographic distribution of NPAs. These parameters could aid deep learning systems in the automatic detection of DR and estimation of its severity.

### 3.3. WF-OCTA and Vascular Reperfusion in DR Eyes

The possibility of vascular reperfusion in DR has been a matter of debate for many years. Some studies suggest that DR may spontaneously regress [50–52], and some older reports claim a rate of reperfusion as high as 69% [53]. It is not yet clear if anti-VEGF agents play a role in this process, and current pieces of evidence are contradictory. Some authors support reperfusion following anti-VEGF agents' injection [54], while others deny any vascular change in response to antiangiogenic treatment [55], despite an overall improvement in the DR severity score.

Couturier et al. noted additional areas of capillary dropouts on WF-OCTA compared to FFA [32]. Additional studies employing WF-OCTA may help in shedding further light on the possibility of capillary reperfusion in treated eyes.

## 4. WF-OCTA and Proliferative Diabetic Retinopathy

**Figure 2:**
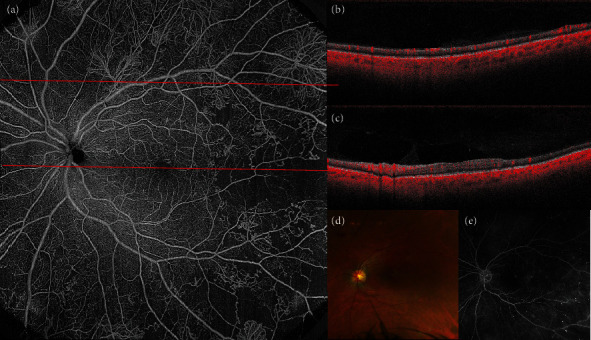
Multimodal imaging of proliferative diabetic retinopathy (PDR). (a) Widefield optical coherence tomography angiography (WF-OCTA) of the posterior pole, revealing multiple neovascularization elsewhere (NVEs) and capillary nonperfusion (NP) temporal to the macula. (b) Structural B-scan passing through the area corresponding to the upper red dashed line. Temporally, NVE is discernible on the B-scan. (c) Structural B-scan passing through the fovea, corresponding to the lower red dashed line and showing temporal retinal thinning secondary to retinal ischemia. (d) Color fundus picture of the same patient, revealing macular exudates, cotton wool spots, and preretinal hemorrhages. (e) Fundus fluorescein angiography shows microaneurysms, capillary nonperfusion, and NVE.

Due to the absence of late dye leakage, OCTA allows a better morphologic characterization of IRMA and NV as compared to FFA (Figure 2).

**Figure 3:**
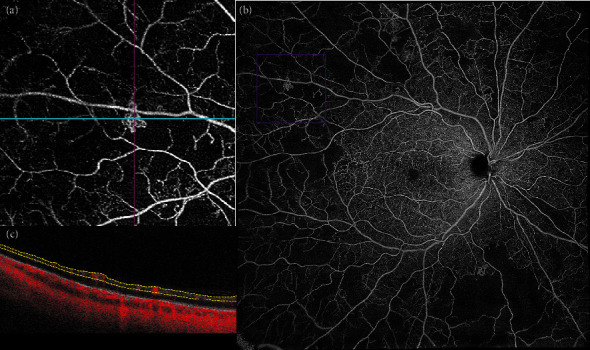
B-scan with slab boundaries and the corresponding flow *en face* image. (a) Widefield optical coherence tomography angiography (WF-OCTA) of the posterior pole, revealing multiple areas of capillary nonperfusion and intraretinal microvascular abnormality (IRMA), outlined in the purple box. (b) Magnification of the area outlined in the purple box, showing IRMA surrounded by capillary nonperfusion. (c) The intraretinal localization of the anomalous vascular network (i.e., the absence of protrusion into the vitreous) makes it possible to differentiate IRMA from neovascularization elsewhere.

IRMAs appear on WF-OCTA as tortuous intraretinal vascular segments not exceeding the inner limiting membrane boundaries; contrarily, neovascularization elsewhere (NVE) protrudes into the vitreous cavity (Figure 3) [19]. Some IRMA may be associated with small vascular tufts, i.e., small buds at their tip with a closed-end and a bulging shape [56]. IRMA shows heterogeneous behavior after panretinal photocoagulation (PRP) treatment: some remain unchanged, some show regression, some others may be worse. IRMA that regresses may be adjacent to areas of restored vascular perfusion after PRP [56].

**Figure 4:**
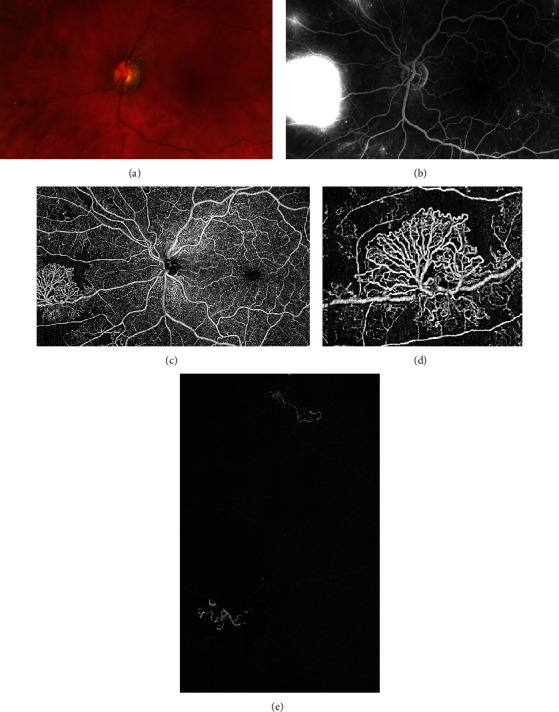
Proliferative diabetic retinopathy with neovascularization elsewhere. (a) Color fundus picture showing the neovascular epiretinal network in the nasal periphery. (b) Fundus fluorescein angiography (FFA) shows marked hyperfluorescence due to dye leakage in the late phase of the exam. Morphologic characterization of the neovascularization is not possible using FFA. (c) Widefield optical coherence tomography angiography of the same eye, depicting the neovascularization (NV) and its peripheral loops (d). (e) Vitreoretinal slab of OCTA, showing the NV protruding into the vitreous.

Both NVE and NV of the disc (NVD) appear as irregular, convoluted masses of large- and small-caliber vessels, better visualized in the vitreoretinal interface (VRI) slab, which covers the most posterior portion of the vitreous body (the hyaloid) and the most anterior part of the retinal surface (Figure 4) [13, 19].

The presence of preretinal vessels can be confirmed by comparing the *en face* OCTA slab with the corresponding OCT B-scan image, which shows flow signal either laying on the retinal surface or protruding into the vitreous cavity in patients with NVE [57, 58]. Structural scans may help in differentiating active from inactive lesions: active NV appears as an exuberant proliferation of fine vessels, while inactive NV features pruned vascular loops of filamentous vessels [58].

Segmentation accuracy and sufficient signal detection are the mainstays for the identification of NV through en face OCTA [59]. Segmentation errors are one of the main sources of artifact in OCTA images [60, 61], and they occur more frequently in NVD than in NVE [59]. Furthermore, the difference in segmentation parameters between multiple SS-OCTA devices must be taken into account when considering the sensitivity of VRI slabs for detecting NVs.

### 4.1. Sensitivity and Specificity of WF-OCTA for NV

A growing body of evidence supports the use of WF-OCTA for the diagnosis and the management of PDR [62]. WF-OCTA may be superior to both indirect ophthalmoscopy and CFP in terms of NV detection [63, 64].

A comparison between UWF-FFA and simulated WF-OCTA in 651 eyes of 433 PDR patients found that the WF-OCTA was able to capture NV in 98% of cases, with a slightly higher sensitivity for treatment-naive eyes (99%) than for treated ones (97%) [62]. Recent studies using different strategies of WF-OCTA montage have confirmed these numbers [34]. WF-OCTA may be particularly advantageous in the case of small neovascular lesions, which could be missed by FFA or misdiagnosed as IRMA or MA.

NVD and NVE have a dissimilar distribution in PDR patients. The majority of the eyes feature both NVD and NVE (50%), 40% display NVE only, and 10% have NVD only [62]. NVE is most prevalent in the superotemporal quadrants and rarely occurs in the nasal quadrants. No significant difference in the NVE/NVD ratio and NVE location has been found between treatment-naive eyes and those that had previously undergone PRP, macular grid laser, intravitreal anti-VEGF injections, or vitrectomy. This might suggest a reevaluation of the clinical significance of NVD in the setting of PDR eyes, which has been traditionally associated with increased severity of the disease. In this view, longitudinal observation is needed to clarify the natural history of NV on WF-OCTA.

The WF-OCTA detection rate of NVE might be limited by NVE located outside the field of view of the WF slab [65]. As predominantly peripheral disease has been associated with faster disease progression [44, 66, 67], adjustments in scan localization (e.g., image centered on the optic disc rather than on the fovea), segmentation, and size are warranted to optimize the sensitivity of noninvasive devices [59].

### 4.2. Role of WF-OCTA in Treated PDR Eyes

To date, only a few studies have investigated the role of WF-OCTA in PDR following PRP or anti-VEGF injections. *En face* OCTA is not inferior to UWF-FFA [68] and represents a reliable tool for defining NV regression, as well as its reactivation and resistance after treatment [69].

**Figure 5:**
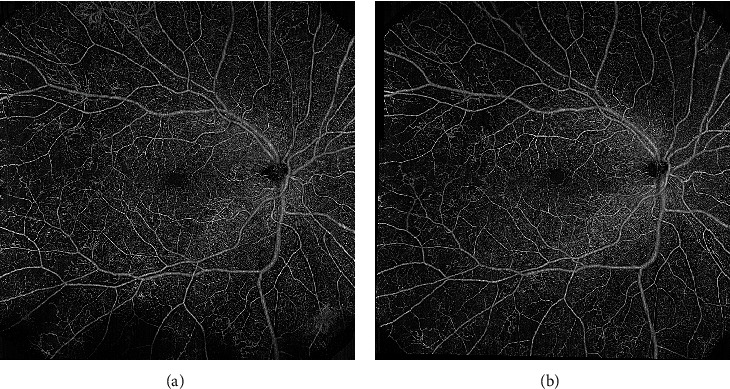
A 12 × 12 mm en face image of the same patient before (a) and after (b) treatment with a combination of panretinal photocoagulation and intravitreal anti-VEGF agents. Complete regression of epiretinal neovascularization is noted. The eyelashes may create masking artifacts (a), which can overestimate the extent of the nonperfusion areas.

Pruning of new vessels and reduction in smaller-caliber vessel density within NV fronds are the most characteristic signs of NV regression, along with a marked reduction of the detected flow area (Figure 5) [58, 68]. Morphologic changes in NV shape and size in regressed NV have been observed as early as 1 week after PRP. Conversely, NVE showing progression at 3 months showed a return to the baseline value in smaller-caliber vessel density at 1 month. Some NVs considered regressed on FFA due to a reduction in dye leakage were deemed as enlarged on OCTA.

## 5. Conclusion

The present review is aimed at exploring, with an evidence-based approach, the usefulness of WF-OCTA in DR patients. WF-OCTA harbors an interesting potential because of its noninvasiveness and high-resolution; moreover, it allows quantitative assessments and comparable metrics of retinal vasculature. Nevertheless, WF-OCTA is still not available in many centers and is susceptible to various types of artifacts. In the coming years, further studies will facilitate the migration of WF-OCTA tools from the research field to everyday clinical practice.

## Data Availability

The data supporting this review are from previously reported studies and datasets, which have been cited. The processed data are available from the corresponding author upon request.
